# Routine RNA-based analysis of potential splicing variants facilitates genomic diagnostics and reveals limitations of *in silico* prediction tools

**DOI:** 10.1016/j.xhgg.2025.100521

**Published:** 2025-09-22

**Authors:** Mark Drost, Jordy Dekker, Federico Ferraro, Esmee Kasteleijn, Marije Verschuren, Evelien Kroon, Hannie C.W. Douben, Inte Vogt, Leontine van Unen, Marianne Hoogeveen-Westerveld, Peter Elfferich, Rachel Schot, Camilla Calandrini, Esther Korpershoek, Frank Sleutels, Hennie B.R. Brüggenwirth, Iris R. Hollink, Lisette Meerstein-Kessel, Lies H. Hoefsloot, Marjon van Slegtenhorst, Martina Wilke, Marjolein J.A. Weerts, Rick van Minkelen, Anja Wagner, Arjan Bouman, Barbara W. van Paassen, Grazia M. Verheijen-Mancini, Ingrid M.B.H. van de Laar, Anneke J.A. Kievit, Judith M.A. Verhagen, Kyra E. Stuurman, Laura Donker Kaat, Marieke F. van Dooren, Marja W. Wessels, Rogier A. Oldenburg, Shimriet Zeidler, Tessa van Dijk, Tahsin Stefan Barakat, Virginie J.M. Verhoeven, Yolande van Bever, Yvette van Ierland, Natalja Bannink, Silvana van Koningsbruggen, Phillis Lakeman, Lisette Leeuwen, Nienke E. Verbeek, Margje Sinnema, Malou Heijligers, Christi J. van Asperen, Jasper J. Saris, Mark Nellist, Tjakko J. van Ham

**Affiliations:** 1 Department of Clinical Genetics, Erasmus MC, University Medical Center Rotterdam, PO Box 2040, 3000 Rotterdam, the Netherlands; 2 Erasmus MC Cancer Institute, Erasmus University Medical Center Rotterdam, Rotterdam, the Netherlands; 3 Department of Pediatrics, Franciscus Gasthuis & Vlietland, Rotterdam, the Netherlands; 4 Department of Human Genetics, Amsterdam University Medical Center, Amsterdam, the Netherlands; 5 Department of Genetics, University Medical Center Groningen, Groningen, the Netherlands; 6 Department of Medical Genetics, University Medical Center Utrecht, Utrecht, the Netherlands; 7 Department of Clinical Genetics, Maastricht University Medical Center, Maastricht, the Netherlands; 8 Department of Clinical Genetics, Leiden University Medical Center, Leiden, the Netherlands

**Keywords:** genetic disorders, diagnostics, RNA splicing, splice prediction algorithms

## Abstract

DNA variants affecting pre-mRNA splicing are an important cause of genetic disorders and remain challenging to interpret without experimental data. Although variant classification guidelines recommend experimental characterization of variant splicing effects, the added value of routine diagnostic investigation of patient mRNA splicing has not been systematically described. Here, we assessed the utility of pre-mRNA splicing analysis in a diagnostic setting for 202 suspected splice-altering variants from individuals referred for genetic testing. Pre-mRNA splicing was assessed in patient cells by RT-PCR, followed by agarose gel electrophoresis and Sanger sequencing and/or exon trapping assays. An effect on pre-mRNA splicing was demonstrated in 63% (*n* = 128/202) of the tested variants. Among the 177 variants initially classified as variants of uncertain significance (VUS), 54% (*n* = 96/177) were reclassified based on pre-mRNA splicing analysis, including 48% (*n* = 85/177) that were upgraded to likely pathogenic or pathogenic. We benchmarked the splice prediction algorithms SpliceAI, SQUIRLS, SPiP, and Pangolin, the tools integrated in Alamut on this clinically relevant and experimentally validated dataset, and the CAGI6 splicing VUS dataset and found variable performance dependent on variant type and location. No single tool classified all variants equally well. We describe several examples of hard-to-predict effects and unexpected results highlighting the limitations of prediction tools, including a not previously described variant type affecting U12-splice site subtype. In summary, we provide a framework for RNA-based analysis in a molecular diagnostic setting, demonstrate the added value of routine testing of RNA from individuals with suspected splice-altering variants, and highlight the limitations of *in silico* prediction tools.

## Teaser

Routine experimental analysis of pre-mRNA splicing enables reclassification of nearly half of the variants of uncertain significance as likely pathogenic. This study highlights limitations of *in silico* mRNA splicing prediction tools and supports integrating RNA-based assays into diagnostics to improve variant interpretation and genetic diagnosis in individuals with suspected splicing-altering variants.

## Introduction

Clinical exome and genome sequencing (ES/GS) have led to the identification of many genetic variants. Our incomplete understanding of the clinical relevance of most of these variants hinders their classification, resulting in many being termed “variant of uncertain significance” (VUS). Clinically, VUSs are problematic because they prevent a definitive genetic diagnosis, complicating accurate prognosis, risk recurrence counseling, prenatal diagnostics, and treatment options.

It has been estimated that >50% of genetic variants that are relevant to disease might cause aberrant splicing of pre-messenger RNA (pre-mRNA).^1^^,^^2^ Unfortunately, the likelihood of aberrant mRNA splicing can be difficult to predict, particularly for variants outside canonical donor and acceptor splice sites. Therefore, in line with guidelines established by the American College of Medical Genetics and Genomics and the Association of Molecular Pathology (ACMG/AMP),^3^^,^^4^ putative spliceogenic DNA variants—variants that affect splicing—often remain classified as VUS. With the increased implementation of clinical sequencing beyond coding exons (GS), it is expected that more (potentially) spliceogenic variants located deep within introns and in other non-coding regions of genomic DNA will be identified. The expected increase in the numbers of spliceogenic VUS necessitates increased understanding of aberrant mRNA splicing. In addition to improving *in silico* predictions, analysis of patient RNA will be important to validate these predictions. For example, a recent study that compared splice predictions across multiple diagnostic centers for 56 clinically and functionally validated variants found that the predictions of at least 50% of the methods used were incorrect for 25% of the tested variants.^5^ In addition, many algorithms do not predict what the effect of the aberrant splicing on the final mRNA transcript will be.^5^^,^^6^^,^^7^^,^^8^ Because of these inaccuracies, experimental testing of mRNA splicing is the best method to determine aberrant mRNA splicing and its outcomes and to facilitate DNA variant classification.^6^^,^^9^ However, experimental mRNA testing is typically not routinely performed in diagnostic laboratories, and standardized procedures and insight into the added value of routine testing of mRNA splicing in comparison to the performance of *in silico* mRNA splicing prediction is lacking.^5^

Here, we analyzed the effects on mRNA splicing of 202 DNA variants identified in individuals with a diverse range of clinical phenotypes who had undergone clinical genetic testing. We performed targeted analysis of mRNA splicing in patient RNA, exon-trapping experiments, or both. In addition, we tested the performance of *in silico* splice prediction tools, including SPiP,^10^ Pangolin,^11^ SpliceAI,^12^ and SQUIRLS^13^ on the identified variants, to assess the overall accuracy of these tools, as well as for specific subsets of variants.

Our analysis shows that routinely assessing mRNA splicing for suspected genetic variants in a diagnostic setting has broad clinical utility, primarily in providing experimental evidence for variant reclassification—mostly VUS to likely pathogenic variants—for half of the variants tested. In contrast, meta-analysis of our clinically relevant, experimentally validated variant cohort together with the CAGI6 splicing VUS challenge cohort with various recently improved splicing algorithms stresses their different limitations, while they have in common that predictions overall provide insufficient evidence for upgrading suspected variants. For example, we show that the occurrence of nonsense-mediated mRNA decay (NMD) and/or of splice abnormalities leading to in-frame deletions are frequent but are poorly predicted *a priori* by *in silico* tools, highlighting the need to perform complementary experimental mRNA analysis, preferably using patient-derived RNA. Finally, we provide examples of disease-causing variants that modulate splicing in unexpected or unconventional ways.

## Material and methods

### Patients, samples, and variant selection

All individuals were evaluated at clinical genetic centers in the Netherlands, a majority at the Erasmus Medical Center in Rotterdam. Pre-test counseling was provided by a clinical geneticist. Informed consent was obtained for diagnostics, including written informed consent from proband and parents for publication of data, in line with the Declaration of Helsinki (institutional review board no. MEC-2012-387). Physicians typically first consulted with the laboratory on whether RNA testing was possible or advisable and what tissue would be required. Typically, an average expression level in “whole blood” or “Cells – Cultured fibroblasts” > 0.1 transcripts per million (tpm) in the GTEx Portal was sufficient for detection by RT-PCR. Where possible, 2 PAX blood samples were collected, as we noticed that the RNA yield after isolation was sometimes low or of insufficient quality, necessitating isolation of RNA from the backup sample.

All variants included in the study were identified by Sanger sequencing, ES, or RNA sequencing (RNA-seq) and were reported back to the requesting clinical geneticist by a laboratory specialist. Variants were annotated according to the Human Genome Variation Society Nomenclature guidelines^14^ and were classified according to the ACMG/AMP criteria.^3^ Variants were mostly flagged as potentially influencing splicing based on Alamut splice predictions; variants were considered potentially spliceogenic when 2 out of the 4 splice prediction algorithms used by Alamut were altered by >10% for a given variant. Analysis of pre-mRNA splicing was subsequently requested by the clinical geneticist, who also counseled the patient on the results of the laboratory testing. Results were discussed in detail by a multidisciplinary team, and where necessary, additional experiments were performed to confirm the effects identified and to determine whether both alleles could be detected. Occasionally, findings were verified by RNA-seq to obtain a more quantitative measurement of the expressed transcripts (e.g., for *BLTP1*, *THOC2*).

### Sample preparation, RNA isolation, and cDNA synthesis

Fibroblasts (from skin biopsies) were grown in Ham’s F10 medium (amniotic cells were cultured in Chang medium) containing 15% (v/v) fetal calf serum and 1% (v/v) penicillin/streptomycin following standard laboratory procedures. Cells were cultured to 70%–80% confluence in 75 cm^2^ tissue culture flasks for the isolation of mRNA. To inhibit NMD, cells were cultured in the presence of 100 μg/mL cycloheximide (CHX) for 24 h prior to mRNA isolation.

Whole blood was collected in PAXgene Blood mRNA tubes (BD Biosciences, Erembodegem, Belgium). mRNA from either blood or tissue was extracted using the RNeasy Mini Kit (QIAGEN, Venlo, the Netherlands). cDNA was generated using the iScript Reverse Transcription Supermix kit (Bio-Rad, Hercules, CA), which combines oligo(dT) and random primers per the manufacturer’s instructions.

### RT-PCR and Sanger sequencing

For each variant tested, primers were designed for PCR amplification of a region containing the variant (primer sequences are available on request). Primers were preferably positioned 2 exons up- and downstream of the variant. Typically, PCR using multiple primer combinations was performed. PCR yield and product sizes were monitored by agarose gel electrophoresis in parallel to control samples and control amplification of a control transcript (*TUBA1A*). PCR products were subsequently analyzed by Sanger sequencing using the BigDye Terminator version 3.1 Cycle Sequencing Mix (Thermo Fisher Scientific, Bleiswijk, the Netherlands), followed by fragment analysis on an ABI3730xl Genetic Analyzer (Thermo Fisher Scientific).

### Minigene exon-trapping system

For exon-trapping experiments, the variant of interest and surrounding sequences, typically ∼400–500 bp, were amplified from genomic DNA and cloned into the pSPL3 vector (Thermo Fisher Scientific, Invitrogen) by Gibson assembly.^15^ Wild-type and variant constructs were verified by Sanger sequencing. We transfected 1 μg of the plasmid using typically 5 μg polyethyleneimine into HEK293T cells growing in a 12-well plate. At 24 and 48 h after transfection, mRNA was isolated and cDNA was generated as described above. Standard primers were used to amplify transcripts derived from the vector. RT-PCR products were verified by agarose gel electrophoresis and Sanger sequencing.

### *In silico* splice predictions

*In silico* splice prediction information was gathered using Alamut Visual Plus (version 1.7; including SpliceSiteFinder-like, MaxEntScan, NNSPLICE, and GeneSplicer). A predicted effect on splicing was defined as ≥2 out of 4 algorithms showing a ≥10% net change in splice site score).

Variants were annotated with Pangolin^11^ version 4.3.1, SPiP^10^ version 2.1, SpliceAI,^12^ and SQUIRLS^13^ version 2.0.1. The pre-computed scores of SpliceAI were obtained from WGSA^16^ version 0.95, and missing annotations were manually filled in using the SpliceAI Lookup webpage (https://spliceailookup.broadinstitute.org/). Further processing was performed in R.^17^ Receiver operating characteristic (ROC) curves were calculated with the R package plotROC^18^ version 2.3.1, precision-recall curves were calculated with the R package yardstic^19^ version 1.2.0, and general statistics regarding the performance of the splicing effect prediction approaches were calculated with the R package caret version 6.0.94.

### RNA-seq analysis

RNA-seq was performed as recently described.^20^

## Results

### RNA splicing analysis reclassifies 54% of VUS

**Figure 1:**
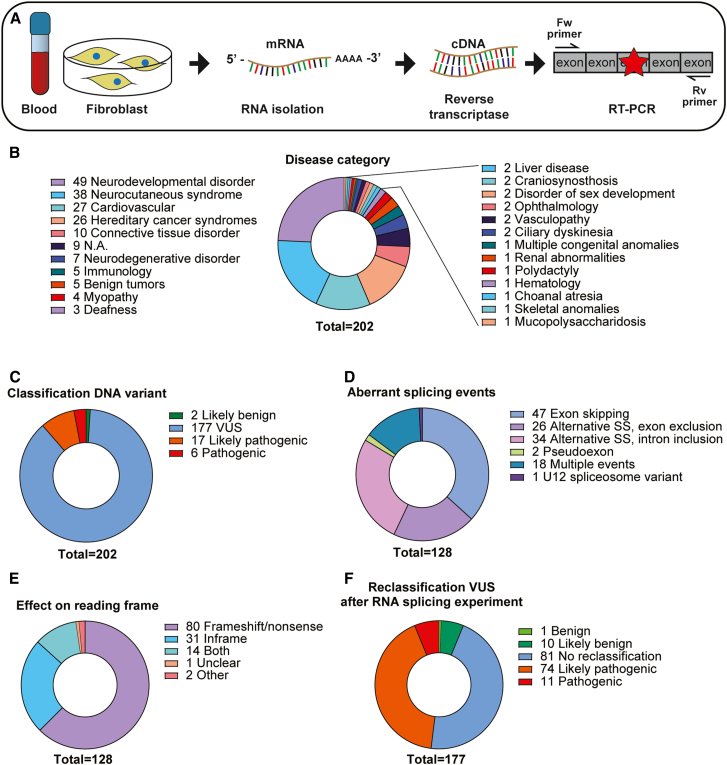
Diagnostic analysis of mRNA splicing identifies various mRNA splicing effects and facilitates DNA variant reclassification (A) Schematic of the experimental setup. Patient tissue is collected (left), mRNA is isolated (second image from the left) and cDNA is generated using reverse transcriptase (second image from the right). A PCR fragment is subsequently amplified using primers that flank the variant (right). mRNA splicing effects are visualized after agarose gel electrophoresis and Sanger sequencing. (B–E) Doughnut plots showing characteristics of the tested DNA variants. (B) Disease categories to which the tested DNA variants were allocated. N.A., not available. (C) Variant classification of the tested DNA variants prior to mRNA splicing analysis. VUS, variant of uncertain significance. (D) Types of aberrant splicing events identified for samples in which aberrant splicing was found (*n* = 128/202). SS, splice site. (E) Effects on reading frame for samples in which aberrant splicing was found (*n* = 128/202). Unclear: in 1 case, the aberrant splicing product could barely be detected; it is likely degraded through NMD. Other: in both cases, the first coding exon of the gene (including the start codon) is skipped. (F) DNA variant reclassification of all VUS tested in this study (*n* = 177/202) after mRNA splicing analysis, combining the mRNA splicing results with other sources of relevant information, including variant frequency, segregation, and clinical phenotype.

Between 2015 and 2023, 202 DNA variants with potential clinical relevance were submitted for experimental testing—in RNA from patients or relatives or using exon trapping assays—for effects on mRNA splicing in our diagnostic laboratory. All variants were identified through routine clinical genetic testing, either by Sanger sequencing, ES, or RNA-seq, prioritized based on factors including variant segregation, population frequency, and phenotypic match, and reported to a clinical geneticist. In most cases (87%, *n* = 176/202), variants in genes relevant for the patient phenotype were identified as potentially spliceogenic by a laboratory specialist (defined as a >10% change in 2 out of 4 of the splice predictions used by the Alamut Visual Plus software package) and subsequent experimental mRNA splicing analysis was requested by a clinical geneticist. In 179 cases, RNA was isolated from tissue, including (1) skin biopsy-derived fibroblasts after culturing in the presence or absence of CHX (an inhibitor of NMD; 40%, *n* = 71/179), (2) amniotic fluid (2%, *n* = 3/179), or (3) from blood collected in PAXgene tubes (58%, *n* = 105/179), including 1 sample of umbilical cord blood) (Figure S1A), and mRNA splicing was analyzed after cDNA synthesis by RT-PCR primers, followed by agarose gel electrophoresis and Sanger sequencing (Figure 1A). In 37 cases, exon trapping was carried out, including 14 cases for which analysis of patient RNA was also performed (Figure S1A). Results were communicated back, with a minimum turnaround time—additional to initial genomic testing—of 2 weeks and a maximum of 4 months.

Within the cohort, variants were in different genes (*n* = 136; Table S1), representing diverse disease groups, including neurocutaneous syndromes (including *NF1* and *TSC1/2*, 24%), neurodevelopmental disorders (19%), cardiovascular disease (13%), and hereditary cancer syndromes (13%; Figure 1B). Of the tested variants, 88% (*n* = 177/202) were classified as VUS based on ACMG/AMP and/or local diagnostic criteria (applied before ACMG/AMP criteria were implemented) prior to mRNA testing (Figure 1C).

We detected aberrant mRNA splicing in 63% of cases (*n* = 128/202; Figure S1C; Table S1). Abnormalities included skipping of a complete exon (37%, *n* = 47/128) and use of an alternative, non-canonical exonic (20%, *n* = 26/128) or intronic (27%, *n* = 34/128) splice site, leading to skipping of part of an exon or to intron retention, respectively. Multiple abnormal splicing events within a single sample (14%, *n* = 18/128), pseudo-exon inclusion (2%, *n* = 2/128), and a U12 splice site subtype switch (<1%, *n* = 1/128; see also below; Figure 1D) were also identified. Aberrant splicing resulted in (predicted) frameshifts (63%, *n* = 80/128), in-frame deletions (24%, *n* = 31/128), or a combination of the 2 (11%, *n* = 14/128; Figure 1E). mRNA splicing results were concordant between RNA isolated from tissue and in exon trapping assays for all 14 variants tested in both systems. In several cases the effect on splicing of a variant was incomplete or, mainly for heterozygous variants, it could not be investigated whether residual normal splicing occurred from the allele containing the variant when no heterozygous coding SNPs were available (Table S1). All results, including the level of abnormal splicing, were subsequently used together with other relevant information, including variant population frequency and clinical phenotype and variant segregation, and often following multidisciplinary case discussions, to reclassify the variants. We were able to reclassify 48% of VUS (*n* = 85/177) as likely pathogenic or pathogenic, thereby providing sufficient evidence for a clinical diagnosis. In addition, 6% of VUS (*n* = 11/177) could be reclassified as likely benign or benign, leading to a combined VUS reclassification rate of 54% (*n* = (85 + 11)/177; Figure 1F).

**Figure 2:**
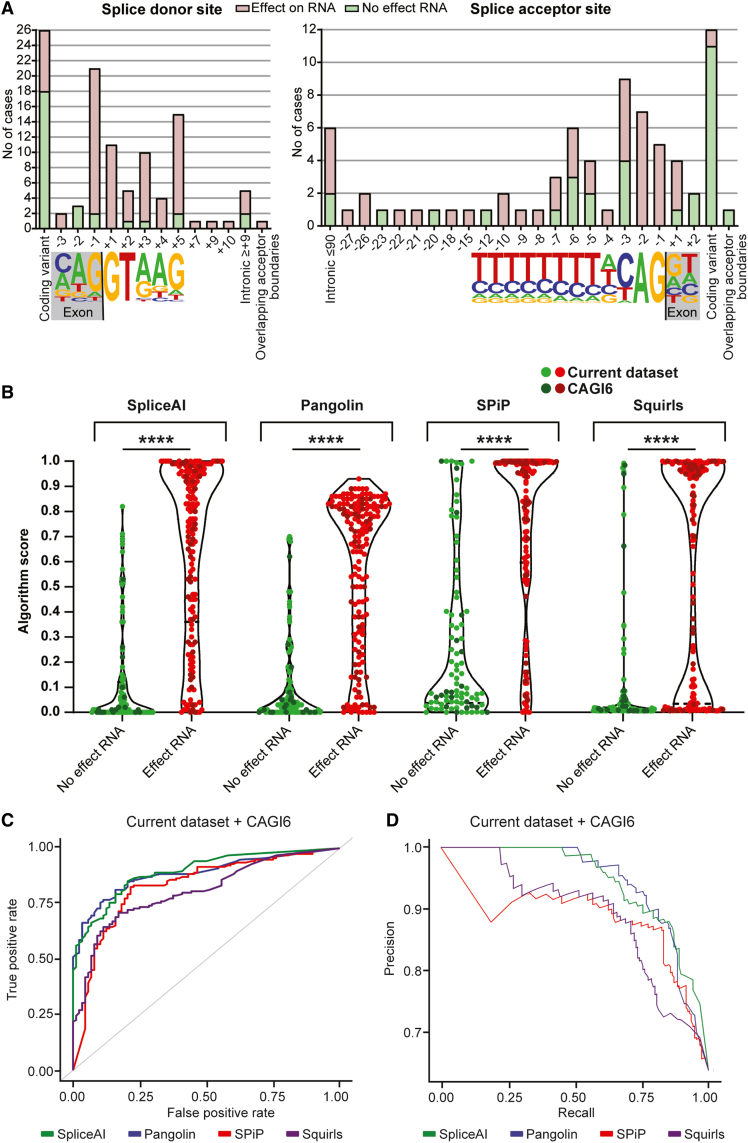
SpliceAI and Pangolin most accurately predict effects on mRNA splicing (A) Bar chart depicting the positions of the DNA variants tested in this study relative to canonical splice donor (left) and splice acceptor (right) sites. Bars are color-coded, depicting the number of variants for which aberrant mRNA splicing was identified. (B) Violin plots showing algorithm scores for Splice AI, Pangolin, SPiP, and SQUIRLS stratified by experimental result (no effect on RNA splicing, in green, and effect on RNA splicing, in red), with each dot representing a single variant from the current dataset (lighter tone) or the CAGI6 dataset (darker tone). ∗∗∗∗*p* ≤ 0.0001, Kruskal-Wallis test with Dunn's multiple comparisons test. (C and D) (C) Area under the receiver operator characteristic curve (AUROC) and (D) area under the precision-recall curve (AUPRC) for Splice AI, Pangolin, SPiP, and SQUIRLS.

The variants tested were often located near exon/intron boundaries, both at the canonical splice donor site (within the last 3 nt of the exon and first 5 nt into the intron; 29%, *n* = 58/202) and the splice acceptor site (within the last 12 nt of the intron and the first 2 nt into the exon; 23%, *n* = 46/202), but a substantial number of tested variants were located outside of these canonical splice sites (49%, *n* = 98/202; Figure 2A). Aberrant mRNA splicing was more often observed for variants within canonical splice sites than for variants outside of canonical splice sites (*n* = 93/116 versus *n* = 26/61, χ^2^ test statistic = 25.5855, *p* < 0.0001; Figure 2A). Consequently, VUS within canonical splice sites were more likely to be reclassified than VUS outside these sites (*n* = 65/98 versus *n* = 23/57, χ^2^ test statistic = 9.9085, *p* = 0.001645; Figures S2A and S2B). Aberrant splicing was observed for all substitutions located at the −1/−2 and +1/+2 positions (*n* = 23).

At least as many intronic variants beyond the +2 donor positions or the −2 acceptor positions influenced mRNA splicing compared to variants that were either exonic or within the +1/+2 donor and −1/−2 acceptor positions (*n* = 63/87 versus *n* = 66/106, χ^2^ test statistic = 3.312, *p* = 0.068776). Strikingly, exonic variants outside of the first and last 2 exonic base pairs had an effect in only 26% of cases. For 58 cases, RNA from fibroblasts treated with CHX was used, and in 23 cases there was an effect on splicing causing a frameshift likely leading to NMD. In 65% of cases (*n* = 15/23) we found a lower level of a frameshift-containing transcript in RNA from non-treated cells compared to RNA from treated cells. Strikingly, in only 1 case did NMD appear to be complete.

Exons differ in their susceptibility for exonic variants to affect splicing,^21^ owing to preexisting optimal or weaker combinations of splicing regulatory elements. We observed that VulExMap categories^21^ correlated with the probability of having an experimentally validated effect on splicing (χ^2^ test statistic = 20.6302, *p* = 0.000126; Table S1). There were fewer exonic variants with an experimentally validated splicing effect in resilient exons (adjusted standardized residual = −2.4692).

### Splicing affecting variants predicted in Alamut can be experimentally validated, but clinically relevant variants are also missed

Most of the variants (*n* = 182/202, 90%) were identified by DNA sequencing and were initially assessed by the splicing prediction algorithms included in Alamut Visual Plus (SpliceSiteFinder-like, MaxEntScan, NNSPLICE, and GeneSplicer). We stratified the predictions of these tools into 2 groups: (1) variants that weakened or strengthened existing or non-canonical 5′/3′ splice sites (SS) and (2) variants with no predicted effect. In 53% (*n* = 36/68) of the variants predicted to create a possible new 5′/3′SS based on a >10% change in ≥2 algorithms, experimental validation did not show evidence for aberrant splicing. In contrast, 97% (*n* = 32/33) of all variants predicted to induce a complete loss of the 5′/3′SS affected splicing, and 75% (*n* = 42/56) of variants predicted to weaken the canonical 5′/3′SS affected splicing. Overall, 68% (*n* = 106/157) variants predicted by Alamut to affect splicing could be experimentally validated. For the remaining 51 variants, we did not find evidence for an effect on splicing.

Alamut did not predict an effect on splicing for 25 variants. Nonetheless, these variants were prioritized for further testing because of a low population frequency, clinical relevance, unexplained RNA-seq results, and/or predictions by other algorithms. For 28% of these variants (*n* = 7/25), we identified an effect on splicing in RNA from the corresponding patient. Importantly, for 71% (*n* = 5/7) of these variants, the experimentally validated effect on splicing was sufficient for reclassification to likely pathogenic or pathogenic. One unconventional variant in *THOC2* affects usage of a U12 minor 3′ acceptor site and is highlighted below.

### Additional splicing prediction tools can help predict variant effect on splicing

Recently, new tools have been developed to predict the effects of DNA variants on pre-mRNA splicing. We assessed the performance of some of these tools on our cohort of experimentally validated, clinically relevant variants. To improve the robustness of our analyses, we merged our cohort (*n* = 202) with the curated variants recently included in the CAGI6 Splicing VUS challenge (a smaller set of clinically ascertained, functionally validated variants; *n* = 56)^5^ and assessed the performance of the *in silico* splice predictions tools SPiP,^10^ Pangolin,^11^ SpliceAI,^12^ and SQUIRLS.^13^ To perform a fair comparison, we focused on a subset of variants that could be scored by all 4 tools (*n* = 243; Table S2), including all the CAGI6 variants.

We evaluated the tools based on their continuous prediction scores. We compared the tools using the area under the ROC curve (AUROC), assessing the relationship between true (TPR) versus false positive rate (FPR), and the area under the precision-recall curve (AUPRC), showing instead the relationship between true positive predictive value (PPV) versus TPR. SpliceAI had the highest AUROC and AUPRC (0.894 and 0.94, respectively; Figures 2B and 2C; Table S3), followed closely by Pangolin (0.885 and 0.942), SPiP (0.830 and 0.89), and SQUIRLS (0.806 and 0.888). Slightly different values were obtained when the analyses were performed on the variants stratified into the 2 individual cohorts, most likely due to the variant heterogeneity between the 2 datasets and to the small sample size (Figures S3C and S3D; Table S3).

In a diagnostic setting, splice prediction tools are often utilized with set thresholds. Therefore, we assessed the performance of the different tools on binary predictions. To do so, we binarized the prediction scores using thresholds obtained from the literature (SQUIRLS: 0.018, SPiP: 0.452, Pangolin: 0.106, and SpliceAI: 0.12). SpliceAI had the highest TPR (0.87; Figure 2D; Table S3), F1 (0.86), and negative predictive value (NPV; 0.76), whereas Pangolin had the highest true negative rate (TNR; 0.77) and PPV (0.86). The PPV and NPV remained relatively unchanged when the SpliceAI cutoff was adjusted to 0.2 (PPV of 0.88, *n* = 84/(84 + 11), and NPV of 0.57, *n* = 54/(54 + 40)). Next, to try to minimize the number of false negatives, we tested the effect of combining prediction tools. We adopted a heuristic approach to investigate the utility of combining the prediction of multiple tools. Assuming a possible effect on splicing when it is predicted by at least 1 tool leads to an increased TPR (0.96), but at the cost of a lower TNR (0.41) and higher FPR (PPV = 0.74). Increasing the minimum number of tools agreeing on the prediction to consider a variant as possibly influencing splicing (at least 2/3/4 tools) led to a progressive increase in the TNR and decrease of TPR.

### Performance by variant type

Variant prediction confidence also depends on the distance of the variant from the nearest splice site.^7^ We investigated how the tools performed when variants were stratified for these positions. We stratified the variants as belonging to the canonical splice acceptor (*n* = 88), the canonical splice donor (*n* = 61; for definition, see above), or variants outside of canonical splice acceptors/donor sites (*n* = 93). We excluded variant NM_007294.3(*BRCA1*):c.5407_5467dup since this variant affected multiple classification bins.

Across the full dataset, SpliceAI had the highest AUROC and AUPRC in all 3 variant stratifications, with the exception of the canonical splice donor site bin, where Pangolin had a marginally higher AUPRC (Figures S3A and S3B; Table S5). We repeated the analyses after setting specific thresholds for each tool (Table S6), as mentioned above for the binary predictions. For the full dataset, we observed that SPiP had the highest TPR at both the canonical splice donor (0.97) and acceptor sites (0.88), whereas SpliceAI had a superior TPR outside of the canonical splice sites (0.89). Pangolin and SpliceAI tied for the highest TNR for the canonical splice donor (0.36) and acceptor sites (0.74), whereas for variants outside of canonical splice acceptors/donors, SPiP had a higher TNR (0.91). Pangolin had the highest PPV for the canonical acceptor site (0.87), tying with SpliceAI for the canonical donor site (0.91). Outside of the splice site, Pangolin had the highest PPV. These results confirm that the performance of these tools is variable and dependent on the position of the variant.

We noticed several important discrepancies between experimental testing and *in silico* splice predictions. Importantly, when using SPiP, SQUIRLS, SPliceAI, Pangolin, or Alamut, respectively, 17%, 20%, 13%, 13%, and 9% variants with effect on splicing (*n* = 128) did not indicate an *in silico* change, thus leading to false negative predictions (Table S2). Conversely, for 32%, 41%, 27%, 26%, and 71% variants where an experimental effect could not be identified (*n* = 74), *in silico* predictions did predict an effect on splicing, suggesting a particularly high FPR for Alamut. Lastly, 4 variants with an effect on splicing were not predicted to affect splicing across all predictors (variants in *TBC1D7*, *BLTP1*, *ALK*, and *MBOAT7*; Table S1).

### Homozygous *CC2D2A* variant causes an acceptor site loss and in-frame deletion

**Figure 3:**
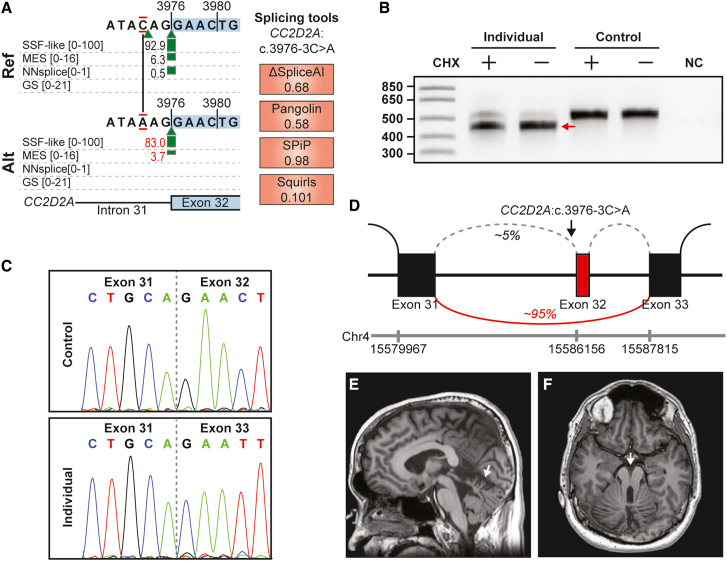
Homozygous in-frame deletion in *CC2D2A* as a cause for Joubert syndrome (A) Alamut splice prediction scores for wild type (top) and NM_001080522.2(*CC2D2A*):c.3976-3C>A, p.(?) (bottom). Note that splice acceptor prediction scores for exon 32 are only moderately decreased for the variant. (B) Agarose gel electrophoresis of RT-PCR products using primers for *CCD2D2A* exons 30 and 34. RT-PCR product size is decreased in the RNA isolated from fibroblasts from individual 1, indicated by the red arrow, both in the presence and absence of CHX treatment. Numerals on the left represent size marker (in nucleotides, first lane). NC, negative control. (C) Sanger sequencing of the amplified RT-PCR products from control (top) and individual 1 (bottom) tissues. Sequence analysis reveals skipping of exon 32 in ∼95% of amplified transcripts. (D) Schematic representation of the effect on mRNA splicing of the NM_001080522.2(*CC2D2A*):c.3976-3C>A variant in the tissue of individual 1. Genomic coordinates for chromosome 4 (GRCh37 (hg19)) are shown. (E and F) Brain MRI of individual 1 depicting a molar tooth sign (white arrows), typical of Joubert syndrome.

For many of the variants in our cohort, we identified mRNA splicing effects, or a lack thereof, that did not correlate with *in silico* splice predictions, but were important for counseling, clinical decision-making, or obtaining a molecular diagnosis. We illustrate several examples below. A homozygous VUS in intron 31 of *CC2D2A*, NM_001080522.2(*CC2D2A*):c.3976-3C>A, p.(?) (gnomAD version 4.1.0 allele frequency 0.00001494, no homozygotes), was identified by ES in a 30-year-old male (individual 1) with intellectual disability. Bi-allelic pathogenic variants in *CC2D2A* cause a range of phenotypes, including severe prenatal onset ciliopathy (Meckel syndrome 6), Joubert syndrome, and late-onset retinitis pigmentosa. Alamut predicted only a marginal reduction in the strength of the exon 32 acceptor site (Figure 3A), whereas SpliceAI predicted its complete loss (Table S1). Analysis of *CC2D2A* transcripts in fibroblast RNA from individual 1 identified nearly complete skipping of exon 32 (Figures 3B–3D), leading to a predicted in-frame deletion of 30 amino acids p.(Glu1326_Asp1355del) within the C-terminal domain. This case therefore supports the strength of SpliceAI *in silico* splice predictions over those in Alamut and shows that by using Alamut predictions only, mRNA splicing effects can be underestimated. To our knowledge, pathogenic truncating and missense variants in this CC2D2A domain have been described,^22^ but no in-frame deletions. Subsequent MRI of the brain of individual 1 revealed a clear molar tooth sign, a hallmark for Joubert syndrome (Figures 3E and 3F), and the variant was upgraded to likely pathogenic.

### Deep-intronic *ATM* variant causes pseudo-exon by likely gain of splice enhancer site

**Figure 4:**
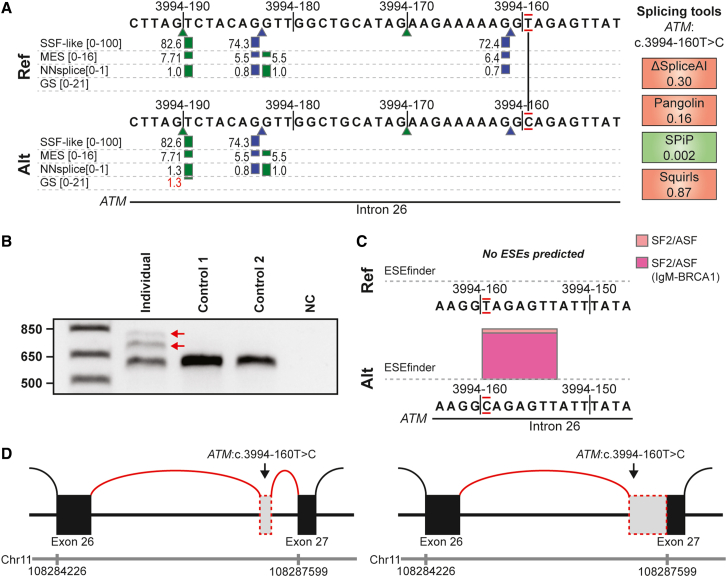
Pseudo-exon inclusion in *ATM* directs therapy choice (A) Alamut splice prediction scores for wild type (top) and NM_000051.3(*ATM*):c.3994-160T>C (bottom). Note the prediction of a non-canonical splice donor at position c.3994-161, which is lost in the variant, and the presence of both a strong non-canonical donor and an acceptor. (B) Agarose gel electrophoresis images after RT-PCR using primers for *ATM* exons 25 and 28. RT-PCR reveals 2 products of increased size in RNA isolated from blood from individual 2, indicated by 2 red arrows. Numerals on the left represent size marker (in nucleotides, first lane). (C) Alamut splice enhancer (exonic splice enhancer [ESE]) prediction for NM_000051.3(*ATM*):c.3994-160T>C (bottom) shows a gain of an SF2/ASF and an SF2/ASF(IgM-BRCA1) ESE. (D) Schematic representation of the effect on mRNA splicing in the blood of individual 2. Genomic coordinates for chromosome 11 (GRCh37 (hg19)) are shown.

In a 67-year-old male (individual 2) with pancreatic cancer and liver metastases, a deep intronic heterozygous variant in intron 26 of *ATM* was identified: NM_000051.3(*ATM*):c.3994-160T>C, p.(?) (absent in gnomAD version 4.1.0). Carriers of pathogenic variants in *ATM* have an increased risk of pancreatic cancer.^23^ The reference allele was predicted to contain a non-canonical splice donor site at the c.3994-161 position, which was absent for the T>C variant allele (Figure 4A). A non-canonical donor (c.3994-183 position) and a non-canonical acceptor (c.3994-190 position) were also predicted to be present, independent of the variant. Analysis of mRNA from individual 2 identified 2 RT-PCR products that were absent from controls (Figure 4B). Sanger sequencing analysis of these products revealed (1) a 112-nt pseudo-exon inclusion (r.3993_3994ins3994-190_3994-79; Figure 4D), likely induced by the creation of a novel exonic splice enhancer (ESE) site (Figure 4C), and (2) retention of the last 190 nt of intron 26 (r.3993_3994ins3994-190_3994-1; Figure 4D). Usage of the c.3994-191 3′SS is also observed in ∼30% of cases in the SpliceVault 300K RNA-seq data, consistent with the idea that such sites are relatively sensitive for variants to influence splicing.^8^ Both RT-PCR products were predicted to lead to a frameshift and premature stop codon in all the detected aberrant transcripts. This illustrates how experimental mRNA splicing analyses can aid in the understanding of deep intronic splicing effects. Considering the RNA analysis, the variant was upgraded to likely pathogenic, thereby supporting the patient’s eligibility for the DRUP (drug rediscovery protocol, https://drupstudy.nl/) study and facilitating therapy choice.

### *BLTP1* heterozygous variant causes loss of an ESE site and exon skipping

**Figure 5:**
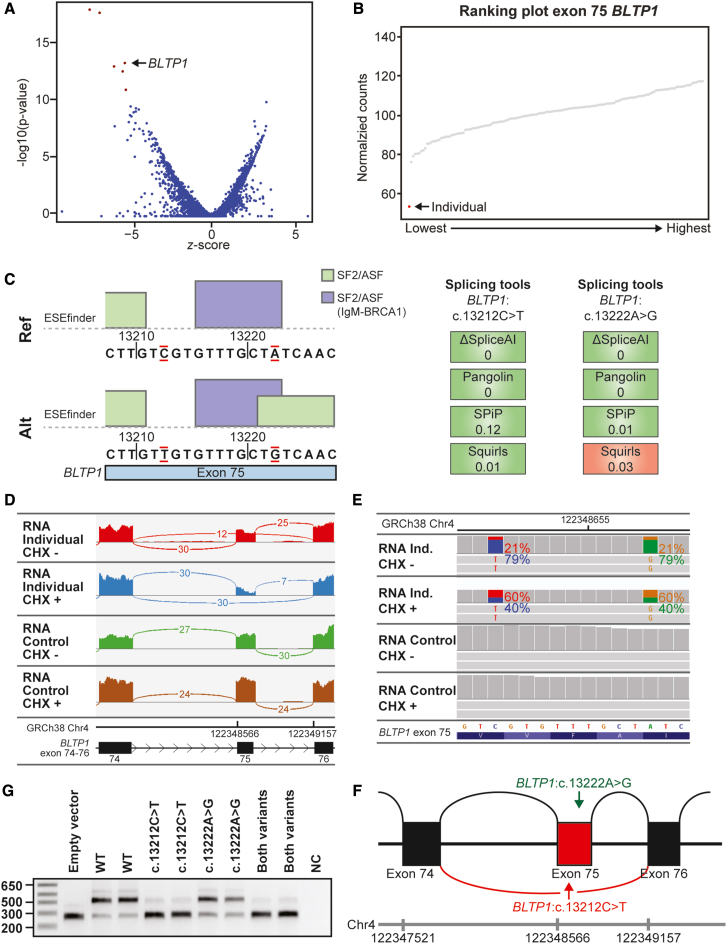
Skipping of *BLTP1* exon 75 through loss of an ESE (A) Volcano plot at gene level (*z* threshold = 4; *p* < 0.0025). *BLTP1* is among the most strongly downregulated genes. (B) *BLTP1* exon 75 ranking plot: individual 5 (red dot, indicated by the arrow, ∼55 counts) has a decreased expression of exon 75 compared to control samples (gray dots, average count ∼100). (C) Alamut ESE prediction for variants NM_015312.3(*BLTP1*):c.13212C>T, p.(Val4404 = ) and NM_015312.3(*BLTP1*):c.13222A>G, p.(Ile4408Val) combined (bottom) shows gain of an SF2/ASF ESE. (D) Integrative Genomics Viewer (IGV) Sashimi plots of RNA-seq data of untreated and CHX-treated samples of individual 4 and a control at the *BLTP1* exon 74–76 region. Note the skipping of exon 75 in the individual’s sample, which is stabilized upon CHX treatment. (E) IGV plot of RNA-seq data (mapped reads) of untreated and CHX-treated samples of individual 4 and a control, showing allelic imbalance at positions NM_015312.3(*BLTP1*):c.13212C>T, p.(Val4404 = ) and NM_015312.3(*BLTP1*):c.13222A>G, p.(Ile4408Val). Calls are skewed toward the maternal transcript (C and A nucleotides, respectively) in the untreated cells, suggesting that exon 75 is skipped from the paternal transcript. (F) Exon trapping assay confirms exon skipping by variant NM_015312.3(*BLTP1*):c.13212C>T but not by NM_015312.3(*BLTP1*):c.13222A>G. Numerals on the left represent size marker (in nucleotides, first lane). (G) Schematic representation of the effect on mRNA splicing in the tissue of individual 4. Genomic coordinates for chromosome 4 (GRCh37 (hg19)) are shown.

A 4-year-old girl (individual 4) was diagnosed with very severe, almost treatment-resistant, epileptic encephalopathy with onset at a very early age, with accompanying severe cognitive impairment (unable to speak), hypotonia, tube feeding because of difficulties swallowing, constipation, and scoliosis. She carried a likely pathogenic, maternal variant, NM_015312.3(*BLTP1*):c.3925C>T, p.(Arg1309∗) (gnomAD version 4.1.0 allele frequency 0.00001239, no known homozygotes) and 2 paternal VUS in exon 75, NM_015312.3(*BLTP1*):c.13212C>T, p.(Val4404 = ) (gnomAD version 4.1.0 allele frequency 0.000007445, no homozygotes) and NM_015312.3(*BLTP1*):c.13222A>G, p.(Ile4408Val) (gnomAD version 4.1.0 allele frequency 0.00001116, no homozygotes). Bi-allelic variants in *BLTP1/KIAA1109* can cause Alkuraya-Kucinskas syndrome (OMIM: 617822), an often-lethal neurodevelopmental disorder characterized by arthrogryposis, brain abnormalities, and global developmental delay, that presents in surviving patients with intellectual disability and epilepsy.^24^ RNA-seq of fibroblast mRNA from individual 4 followed by *Z* score-based expression outlier detection showed *BLTP1* as one of the most strongly divergent transcripts (Figure 5A),^20^ with exon 75 of the *BLTP1* transcript reduced to ∼50% of control levels (Figure 5B). *In silico* tools did not predict an effect on mRNA splicing (Table S1), but a novel SF2/ASF splice factor type ESE binding site was predicted by ESEfinder^25^ (included in Alamut) to be introduced by variant NM_015312.3(*BLTP1*):c.13222A>G, p.(Ile4408Val) (Figure 5C). The balance between exonic splicing silencers (ESS) and ESEs strongly influences whether a pre-mRNA sequence is included as an exon in a mature transcript.^2^ Inspection of the RNA sequencing data showed skipping of *BLTP1* exon 75 from the paternal allele causing a frameshift transcript (r.13130_13280del, p.(Asp4377Valfs∗7)) (Figure 5E), which was stabilized upon CHX treatment (Figure 5D). Based on the RNA-seq data, we could not precisely determine whether the skipping of exon 75 was complete, as transcripts from both alleles may undergo NMD. However, as complete *BLTP1* loss of function was reported to be incompatible with life, it is likely that a paternal pathogenic variant would have an incomplete effect.

We next assessed what the individual contributions of both paternal variants were to exon 75 splicing using mini-gene exon trapping experiments. This revealed that variant NM_015312.3(*BLTP1*):c.13212C>T but not variant c.13222A>G caused enhanced skipping of exon 75 (Figures 5F and 5G), leading to the reclassification of variant NM_015312.3(*BLTP1*):c.13212C>T as likely pathogenic and confirming the molecular diagnosis. This shows how mRNA splicing analyses, including mini-gene exon trapping experiments, can help distinguish the effects on mRNA splicing when multiple variants that may affect mRNA splicing are present within a single exon.

### Hemizygous *THOC2* variant causes a U12 splice site donor/acceptor combination subtype switch

**Figure 6:**
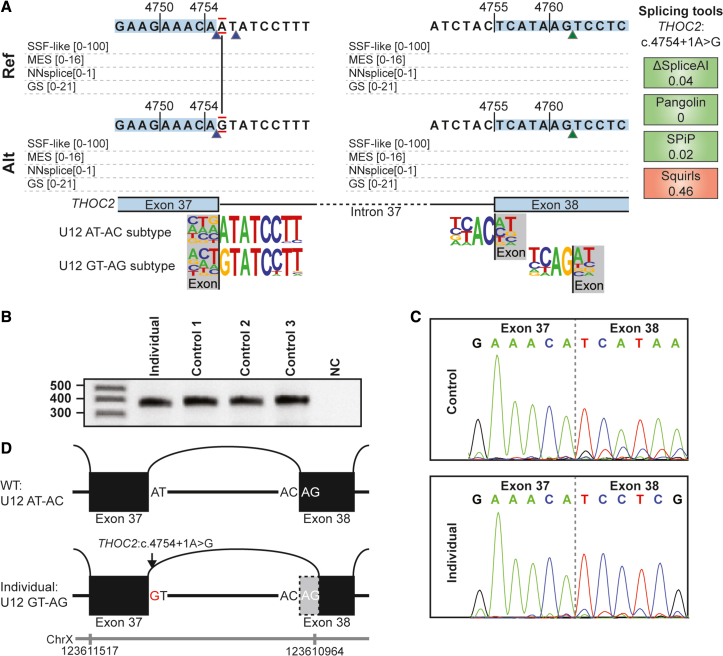
U12 splice site donor/acceptor combination subtype switch induced by a splice donor variant in *THOC2* (A) Alamut splice prediction scores for wild type (top left and right) and NM001081550.1(*THOC2*):c.4754+1A>G (bottom left). This variant is located at the intron 37 splice donor. The intron 37 splice acceptor is also depicted to illustrate its sequence context in relation to the U12 subtypes (bottom). (B) Agarose gel electrophoresis images after RT-PCR using primers for *THOC2* exon 35 and the 3′ UTR. Numerals on the left represent size marker (in nucleotides, first lane). (C) Sanger sequencing of the amplified RT-PCR products from control (top) and the blood of individual 3 (bottom) reveals a 7-nt deletion at the exon 38 splice acceptor. (D) Schematic representation of the effect on mRNA splicing in the blood of individual 3. Genomic coordinates for chromosome X (GRCh37 (hg19)) are shown.

In a 3-year-old male (individual 3) hemizygosity for the variant NM_001081550.1(*THOC2*):c.4754+1A>G, p.(?) was identified in exon 37 (out of 38). THOC2 is a subunit of the TREX complex, important for nascent mRNA transport, and pathogenic *THOC2* variants can cause an X-linked intellectual developmental disorder.^26^ The canonical splice donor site of exon 37 is poorly predicted by Alamut (Figure 6A), likely because this is not a canonical, major U2 intron but a minor spliceosome-type U12. Due to the uncommon U12 splice donor site, the clinical phenotypic match with individual 3 and *THOC2-*related disorders, and absence of the variant from healthy controls, *THOC2* splicing was analyzed in mRNA isolated from the blood of individual 3. Agarose gel electrophoresis (Figure 6B) followed by Sanger sequencing (Figure 6C) revealed almost complete use of an alternative acceptor splice site in exon 38 leading to a frameshift, stop loss, and predicted 7-amino acid extension of the normally 1,593-amino acid protein NM_001081550.1(*THOC2*):r.4757_4763del, p.(His1586Profs∗14) (Figure 6D). This reveals that even when canonical splice sites are not accurately predicted by Alamut, an effect on mRNA splicing by a variant at such a position cannot be ruled out. An explanation for this unexpected result is that there are 2 U12 acceptor-donor combinations.^27^^,^^28^ The first U12 splice site combination consists of a splice donor site with an AT at position +1/+2 and a splice acceptor site with an AC at position −1/−2 (referred to as an AT-AC splice site), whereas the second combination consists of a GT-AG splice donor and acceptor site. The c.4754+1A>G variant changes the U12 splice donor site from an AT to a GT, resulting in an AT-AC to GT-AG subtype switch, to the non-canonical splice U12 AG splice acceptor site in exon 38 (Figure 5A). Thus, the c.4754+1A>G variant causes a U12 subtype switch, leading to utilization of an alternative splice acceptor site and resulting in a frameshift. Because the frameshift occurs in the last exon of *THOC2*, the aberrant transcript is not predicted to undergo NMD.^4^ We presume this variant underlies the clinical phenotype in individual 3.

## Discussion

Here, we assessed mRNA splicing for 202 potentially clinically relevant spliceogenic variants, for which diagnostic testing was requested. Prior to testing, 88% of them were classified as variants of unknown clinical significance, and many were initially selected due to a predicted effect on splicing according to Alamut. Our experiments led to reclassification for 54% of the variants, mostly upgraded to likely pathogenic or pathogenic. This led to a definitive diagnosis, facilitating therapy choice, reproductive options, or presymptomatic testing of family members. Meta-analysis of our cohort merged with the CAGI6^5^ cohort of curated, clinically relevant variants showed that of the 4 tested recently developed splice prediction tools SQUIRLS, SPiP, SpliceAI, and Pangolin, the last 2 performed best overall. Of note, SpliceAI had the highest AUROC/AUPRC, TPR, F1, and NPV, whereas Pangolin performed best on TNR and PPV. All 4 tools led to substantial false negatives/positives, and none performed best for all categories. Although we did not include Alamut in our extensive analysis, we note that the PPV of the tools in Alamut (68%) is lower than the more recent tools included in our analysis. Furthermore, for the variant cohort described here, the highest TPR achieved by a single tool is that of SpliceAI (0.87), while Pangolin had the highest PPV (0.87). We also show that combining predictions from multiple tools can further improve TPR and/or PPV, depending on the specific parameters that are applied. Importantly, the capacity to accurately predict clinically relevant spliceogenic variants that often go undetected—such as deep intronic variants—is not well established. Therefore, even though splice prediction tools are effective at recognizing most relevant candidate spliceogenic variants, all variants will require experimental testing to establish their effects and, in combination with other genetic and clinical evidence, provide sufficient evidence to support a molecular diagnosis.

Recent studies report the added value for *in vitro* testing of potentially clinically relevant, spliceogenic variants and suggest that RNA analysis should routinely be considered in genetic disease diagnostics.^6^^,^^9^ Wai et al.^9^ tested 257 variants in RNA from blood directly surrounding the 5′/3′SS. Abnormal splicing was identified in 33% of cases, and SpliceAI performed better overall than Alamut in predicting which variants might have an effect. Bournazos et al.^6^ tested 74 variants surrounding the 5′/3′SS and focused on standardized practices for PCR-based RNA diagnostics and to use the results from mRNA splicing experiments as supporting evidence for ACMG variant classification. In contrast to these studies, for the 202 variants described here, diagnostic testing was requested by the attending clinical geneticist and carried out in a diagnostic setting. Unlike most splicing studies that used strict criteria for variant inclusion and tested mainly high-risk variants in 5′/3′SS for which splicing predictions typically work well, ascertainment criteria of our variants here were relatively loose and driven by a clinical question. Results were discussed in weekly meetings with technicians and laboratory specialists and weighed with available evidence, including clinical phenotype, disease mechanism, variant segregation, and population frequencies. Support for reclassification of 54% of the variants, from VUS to likely pathogenic, was obtained, showing the potential impact that routine RNA-based analyses could have in diagnostic laboratories. Our variant set was more heterogeneous than in previous studies, as we tested many clinically relevant variants outside the canonical 5′/3′SS, including coding variants, deep intronic variants, variants affecting splice enhancers, and 1 likely pathogenic variant causing a switch in the use of a U12 5′/3′SS combination that has not previously been reported to our knowledge. With increased clinical GS, the number of relevant spliceogenic variants is likely to increase significantly.

An important question is whether RNA-seq could be used as a more standardized test to detect the effects of the variants described here, instead of bespoke RT-PCR requiring custom primer sets and Sanger sequencing of cDNA from multiple tissue sources.^20^^,^^29^ The main advantage of RT-PCR is that ∼50-fold lower levels of mRNA expression can still be detected with routine parameters (e.g., *ALPK3*, *DAB1*, and *SHANK2* in fibroblasts; *SLC45A2*, *CLCN4*, and *RAD51C* in blood) compared to RNA-seq where typically a median ∼5 tpm is required.^8^^,^^20^ In our current cohort almost half of the genes have a tpm below 5 and are therefore unlikely to have sufficient coverage in RNA-seq datasets. RNA-seq could in principle be a faster, more robust test, but this typically relies on other factors, including where the sequencing is performed (in-house/outsourced), what batch sizes are needed, and storage and computing capacity. For example, cDNA synthesis, PCR, and Sanger sequencing can be performed directly after RNA isolation, even for single samples, whereas for RNA-seq, larger batches are processed at once.

Recent work has showed that some exons—vulnerable exons—are more often affected by exonic splicing variants due to intrinsic characteristics, including a weaker exon definition, as they contain weaker 5′/3′SS, fewer putative exonic splicing enhancers, and/or more silencers.^21^ This makes them more sensitive to variants affecting regulatory sequences that would cause aberrant splicing. Our analyses and data are in line with these observations, as we noted fewer exonic variants with a validated effect on splicing among variants falling within resilient exons when compared to variants in vulnerable exons, while variants were more often spliceogenic within ±3 bp of the exon-intron boundaries.

We chose to use different tissue types depending on expression levels in the GTEx Portal,^30^ and, when applicable, the most appropriate tissue type for the individual and/or family members undergoing testing. Of note, transcript isoforms exist with expression levels that can differ among tissues, and the most relevant tissues for a given disorder may not be accessible for testing. For this reason, effects of variants tested could be over- or underestimated. We have accounted for overestimation because, in terms of variant (re)classification, evidence types other than effects on mRNA splicing alone are incorporated. Alternatively, if no effect on mRNA splicing was observed, the variants were typically classified as VUS rather than likely benign, to avoid underestimating their potential impact.

PAXgene blood RNA proved particularly useful when a fast result was needed (e.g., because of a pregnancy), and as RNA could be directly extracted the same day, a diagnostic turnaround time of ∼2 weeks could be reached. As described, heterozygous coding SNPs typically helped to establish phasing and the nature of the effect of a (non-coding) heterozygous spliceogenic variant in RNA from blood.^6^ Splicing changes leading to a frameshift often lead to NMD and, potentially, misinterpretation of the result as a lack of an effect on splicing. Cultured fibroblasts or amniocytes were used in 74 cases, and CHX treatment in 15/23 cases where a stop codon was introduced assisted in detecting transcripts that were subject to NMD. Strikingly, even though a premature stop occurred before the last 2 exons, in only 1 case did NMD appear to be complete, and in 35% of cases (*n* = 8/23) NMD could not be detected at all. Although in some of these it is possible that a truncated (but potentially active) protein could still be made, other mechanisms preventing NMD are also possible (e.g., a lack of nuclear transport).^31^ Although it took on average 5 weeks to culture fibroblasts from skin biopsies, cryopreserve cells, and obtain RNA, the quality of the RNA was typically better and more consistent than RNA from PAX tubes.^20^ Indeed, analyses in fibroblasts led to reclassifications more often (*n* = 42/71, 59%) compared to analyses in blood (51%, *n* = 53/104).

In 23 cases we performed exon trapping. This was for 3 main reasons: (1) when a relevant tissue with sufficient, detectable expression could not be obtained—for example, because the median tpm, as indicated in the GTEx Portal, was too low or too variable (e.g., *ALK*, *RYR1*, *MYH6/8*, *SLC6A1*); (2) to provide supporting evidence for an effect on the splicing of a variant, where the effect on splicing was found first by RNA-seq (e.g., splice enhancer mutations in *BLTP1*, *AIFM1*, *TBC1D7*, and *MFSD8*) and splice predictions were insufficient to support a causal role of the variant^20^; and (3) when the effect on splicing was uncertain based on tissue RNA, often due to the lack of heterozygous SNPs to phase effects on splicing.

Gains of 5′/3′SS inside exons were frequently identified by Alamut-based selection of potentially spliceogenic variants and were classified as VUS. In our cohort, 53% of these SS gains did not affect splicing, and of the remainder, only a few had an effect sufficient for reclassification. In contrast, of the splice variants causing weakened 5′/3′SS, many fewer had no effect (25%). Furthermore, 7 variants did not show a strong change in any of the tools included in Alamut but showed an effect sufficient for reclassification. The capacities to detect such variants were overall better for SPiP, SQUIRLS, SpliceAI, and Pangolin. Thus, 5′/3′SS gains as predicted by Alamut are often false positives. Our results suggest that such splice site gains are more likely to affect splicing and support pathogenicity, if they become stronger than the most nearby canonical splice site (17/28; 60%) than if they are weaker (1/24; 4%). In contrast, SPiP, SQUIRLS, SpliceAI, and Pangolin also take the nearby sequence context into account, leading to fewer false positives.

Importantly, the effect of a variant on mRNA splicing, even if pathogenic, is often incomplete. Although thresholds for abnormal splicing have been suggested in the context of ACMG criteria,^6^ we did not use specific criteria for levels of abnormal splicing. Results were considered on a case-by-case basis, considering all relevant sources of evidence available. In several cases, although the level of abnormal splicing was relatively small, we upgraded the variant to likely pathogenic, and the argumentation differed per case. For example, for the *BLTP* variant, the incomplete effect fits the genotype-phenotype correlation as full loss of function is not compatible with life. In case of variant NM_020774.3(MIB1):c.1677G>C, p.(Gln559His), which shows only ∼50% abnormal splicing for the allele with the variant, the missense variant itself is very likely to perturb protein function. Other indirect evidence includes the fact that the *MIB1* gene contains very few benign missense variants, that the variant is not in gnomAD, and that there is a strong phenotypic match with the patient. Similarly, 7 variants in *NF1* have an incomplete effect, but due to the recognizable and specific neurofibromatosis phenotypic features, low variant frequency, and lack of other relevant variants in *NF1*, these were considered likely pathogenic.

We previously highlighted the effect of variants with unconventional or unexpected effects. This included in-frame deletions—for example, where a VUS was prioritized (the previously described *NF1*:c.2710T>A, p.(Cys904Ser), amino acid substitution which did not affect NF1 function).^32^ However, in patient RNA, a transcript encoding an in-frame deletion, p.(Cys904_Val951del), due to the creation of a non-canonical donor site, was identified. Functional testing demonstrated that the NF1 p.Cys904_Val951del variant protein was deficient in an NF1-SPRED1 coimmunoprecipitation assay.^32^ Similar are our findings in an individual with a homozygous *CC2D2A* variant causing an in-frame deletion and a clinical phenotype that appeared non-typical for Joubert syndrome—only to be confirmed at MRI examination by the characteristic molar tooth pattern of cerebellar and brainstem malformation.^33^

Variants that affect splice enhancers or cause pseudo-exons are difficult to predict *a priori*. For example, the *BLTP1*:c.13212C>T variant caused exon skipping possibly due to increased splice suppressor binding, as the variant increases the ESS/ESE ratio from 20/49 = 0.41 to 28/49 = 0.51. Unfortunately, many variants affect this ratio and do not affect splicing, and therefore this information is *a priori* insufficient to predict an effect on splicing. We previously described 3 other variants that cause skipping by affecting the ESS/ESE ratio, and these variants have little in common.^20^ In contrast, we hypothesize that the deep-intronic *ATM* variant activates a pseudo-exon by introducing a splice enhancer site. The *THOC2* variant affecting the U12 donor/acceptor combination choice is unprecedented to our knowledge and highlights the possibility that many more spliceogenic variants reside in the genome and may already be detectable by ES but may fail to be prioritized by standard diagnostic analyses and require experimental validation in patient RNA. Strikingly, as the type of variants currently identified by DNA sequencing versus those identified primarily by RNA-seq show little overlap, the number of pathogenic splicing variants is likely underestimated.

Importantly, in cases where splicing analysis leads to reclassification to a likely pathogenic or pathogenic variant, this is often the end of a long diagnostic odyssey. Additionally, expensive laboratory tests are no longer required, and unnecessary monitoring and treatment can be terminated. In almost half of the individuals tested the variant was reclassified to likely pathogenic or pathogenic, providing essential evidence to obtain a molecular diagnosis. Although the precise clinical impact for such a diverse cohort is difficult to measure, it includes improved care, screening of family members, prenatal testing, and sometimes therapy. For example, in the individual with the *UNC13D* variant that was reclassified after splicing analysis to a likely pathogenic variant, a hematopoietic stem cell transplantation was carried out; and an individual with pancreatic cancer and an *ATM* variant that was reclassified to likely pathogenic upon analysis of splicing, was included for experimental treatment. Furthermore, prenatal testing and preimplantation genetic testing is possible in families harboring variants in *NF1* and *TSC1* variants. Routine diagnostic analysis of mRNA splicing is therefore an effective approach, with broad clinical utility to proceed with spliceogenic VUS, leading to a high diagnostic yield for a wide range of clinical disorders and improved care for individuals and families with genetic disorders.

## Data and code availability

This study did not generate/analyze code.

## Acknowledgments

We thank the patients and their families for their willingness to contribute to this study, with the aim of improving diagnostic analysis of splicing variants in genetic disorders. We thank Gideon Huigen, Roy Lamping, Noach Minderhoud, Lot van Ulft, and Lida Prins-Bakker for RNA isolation and cell culturing experiments. We thank Nicole van Koetsveld, Annemieke Trebitsch, Ayse Sener, Sally den Boer, Chantal Elling, Cindy Becht, and Jamilieh Hosseini for culturing fibroblasts. We thank Gideon Huigen, Roy Lamping, Martine van Amelsvoort, Lida Prins, and Chérise Jurriens for performing the RNA isolations.

## Author contributions

Conceptualization, M.D., J.D., F.F., and T.J.v.H.; methodology, M.N., J.J.S., M.D., J.D., F.F., E. Kasteleijn, H.C.W.D., L.v.U., M.H.-W., E. Kroon, P.E., R.S., and I.V.; formal analysis and investigation, all authors; writing – original draft preparation, M.D., J.D., F.F., and T.v.H.; writing – review & editing, M.D., J.D., F.F., J.M.A.V., T.S.B., V.J.M.V., M.N., and T.v.H.; supervision, M.N. and T.v.H. All authors read, commented, and approved the final manuscript.

## Declaration of interests

The authors declare no competing interests.

## Web resources

gnomAD, http://gnomad.broadinstitute.org
GTEX Portal, https://gtexportal.org/home/
Integrative Genomics Viewer (IGV) web application, https://igv.org/app/
OMIM, http://omim.org/
OUTRIDER, https://github.com/gagneurlab/OUTRIDER
Pangolin, https://github.com/tkzeng/Pangolin
SPiP, https://github.com/LBGC-CFB/SPiP
SpliceAI, https://spliceailookup.broadinstitute.org/
SQUIRLS, https://github.com/monarch-initiative/Squirls
Voila application, https://github.com/voila-dashboards/voila
Web browser application for RNA-seq analysis, https://github.com/KlinGenErasmusMC/rnaseq-voila

## Supplemental information

Data S1. Tables S1–S6

Document S2. Article plus supplemental information
