# Supplementary material for: Routine RNA-based analysis of potential splicing variants facilitates genomic diagnostics and reveals limitations of in silico prediction tools
Source: HGG Adv. 2025 Sep 22;7(1):100521. doi: 10.1016/j.xhgg.2025.100521 (PMC12547740; doi:10.1016/j.xhgg.2025.100521)
Supplement: Document S1. Figures S1–S3 [file mmc1.pdf]

HGGA, Volume 7

Supplemental information

Routine RNA-based analysis of potential splicing variants facilitates genomic diagnostics and reveals limitations of *in silico* prediction tools

Mark Drost, Jordy Dekker, Federico Ferraro, Esmee Kasteleijn, Marije Verschuren, Evelien Kroon, Hannie C.W. Douben, Inte Vogt, Leontine van Unen, Marianne Hoogeveen-Westerveld, Peter Elfferich, Rachel Schot, Camilla Calandrini, Esther Korpershoek, Frank Sleutels, Hennie B.R. Brüggenwirth, Iris R. Hollink, Lisette Meerstein-Kessel, Lies H. Hoefsloot, Marjon van Slegtenhorst, Martina Wilke, Marjolein J.A. Weerts, Rick van Minkelen, Anja Wagner, Arjan Bouman, Barbara W. van Paassen, Grazia M. Verheijen-Mancini, Ingrid M.B.H. van de Laar, Anneke J.A. Kievit, Judith M.A. Verhagen, Kyra E. Stuurman, Laura Donker Kaat, Marieke F. van Dooren, Marja W. Wessels, Rogier A. Oldenburg, Shimriet Zeidler, Tessa van Dijk, Tahsin Stefan Barakat, Virginie J.M. Verhoeven, Yolande van Bever, Yvette van Ierland, Natalja Bannink, Silvana van Koningsbruggen, Phillis Lakeman, Lisette Leeuwen, Nienke E. Verbeek, Margje Sinnema, Malou Heijligers, Christi J. van Asperen, Jasper J. Saris, Mark Nellist, and Tjakko J. van Ham

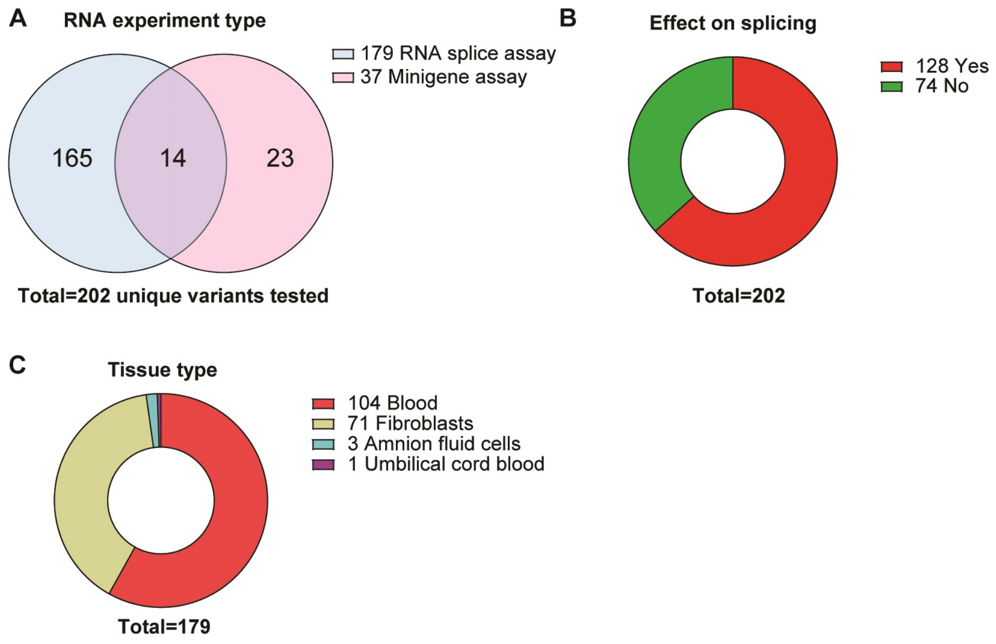

Figure S1. Additional characteristics of mRNA splicing experiments performed in this work.

A. Venn diagram, illustrating the mRNA splicing experiment type used for each tested variant. **B-C.** Doughnut plots showing: **B.** Fraction of DNA variants for which an effect on mRNA splicing was observed. **C.** Tissue types from which RNA was isolated for this study.

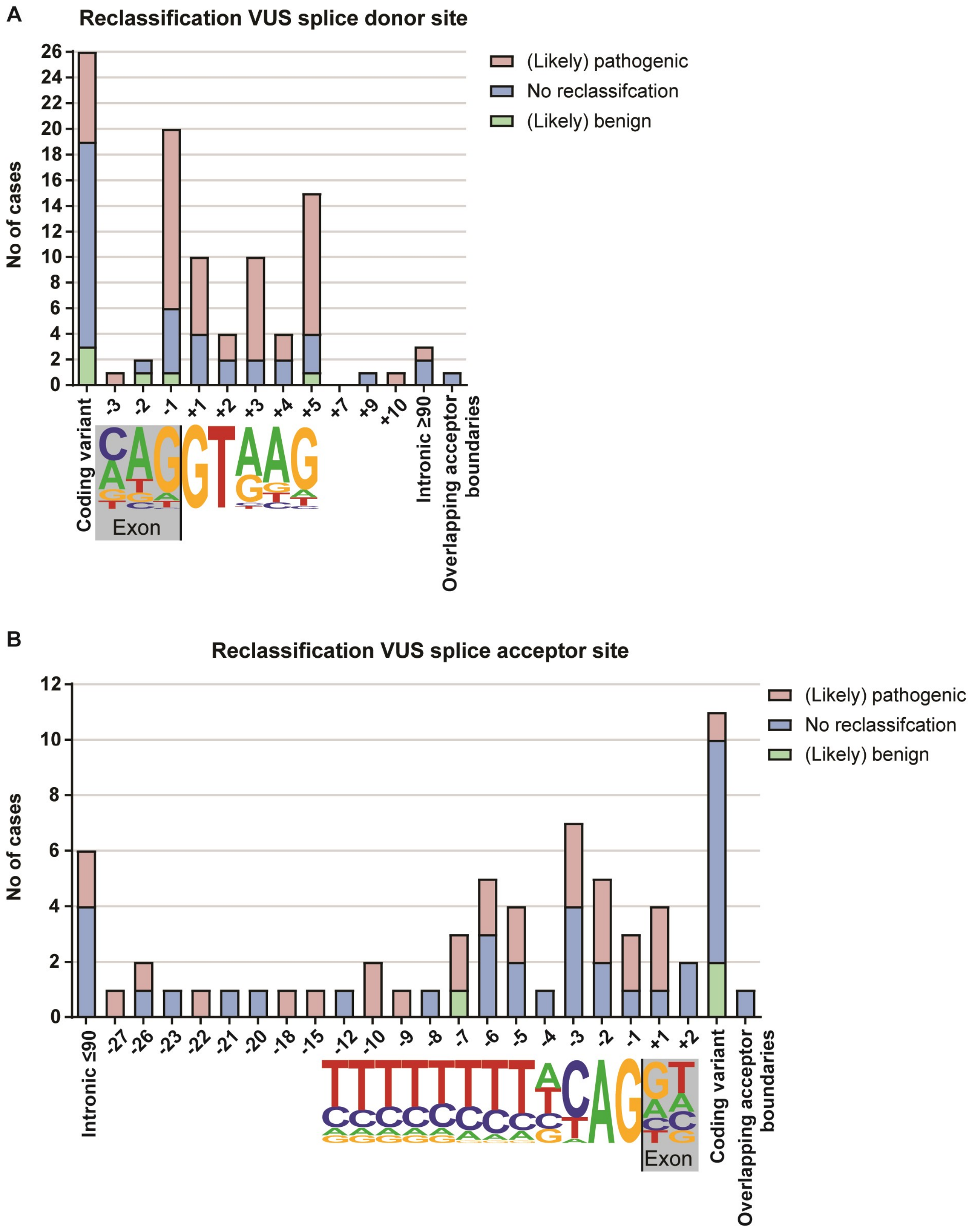

Figure S2. Variants in/proximal to the canonical splice donor/acceptor sites are more often reclassified than those on more distal positions.

A. Bar chart depicting the positions of VUS tested in this study relative to the canonical splice donor site. Bars are color coded, reflecting the number of variants that were reclassified after experimental mRNA splicing analysis and their final classification. **B.** As in A, but for the canonical splice acceptor site.

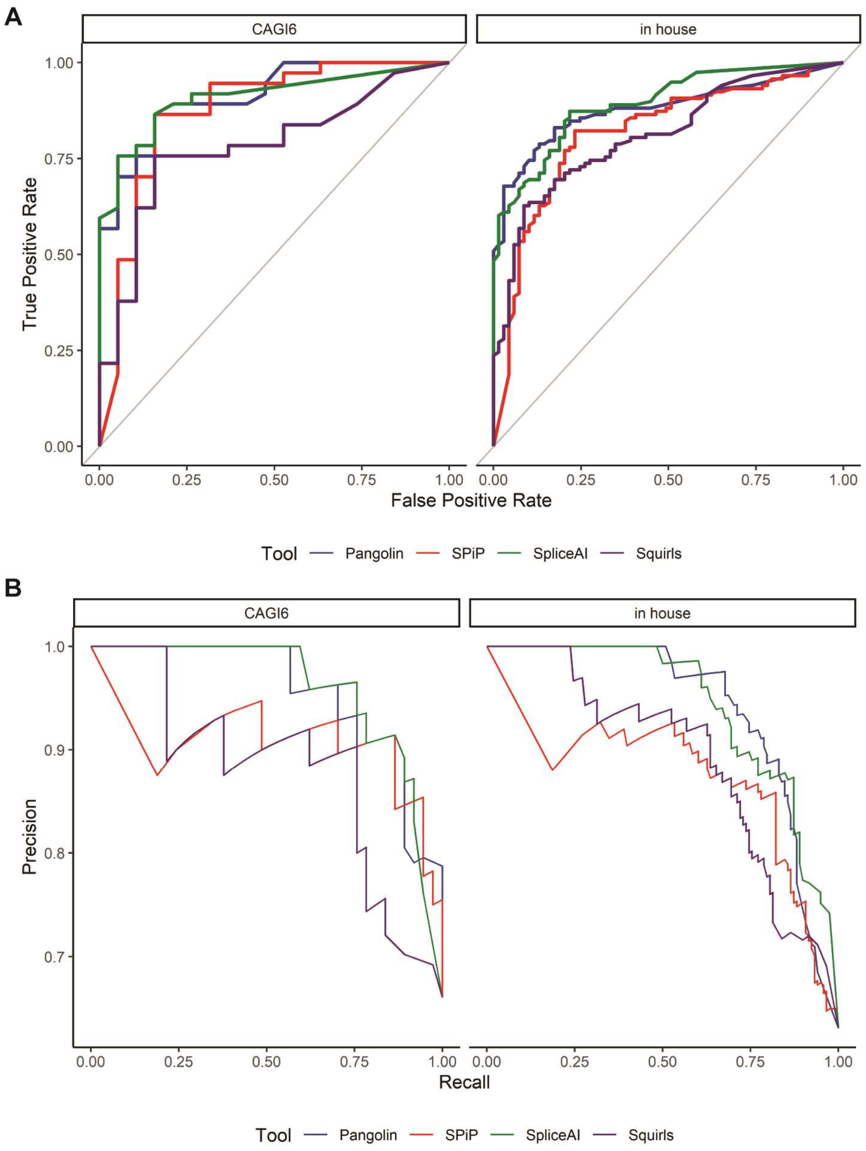

Figure S3. SpliceAI and Pangolin most accurately predict effects on mRNA splicing also based on individual, rather than merged, datasets.

A. Receiver Operator Characteristic curve (AUROC) and **B.** Precision-recall curve (AUPRC) for Splice AI, Pangolin, SPiP and Squirls, similar to Figure 2B and C, but now stratified based on the individual datasets used (CAGI6 to the left, in house set to the right).
